# On the use of the not‐at‐random fully conditional specification (NARFCS) procedure in practice

**DOI:** 10.1002/sim.7643

**Published:** 2018-04-02

**Authors:** Daniel Mark Tompsett, Finbarr Leacy, Margarita Moreno‐Betancur, Jon Heron, Ian R. White

**Affiliations:** ^1^ MRC Biostatistics Unit Cambridge Institute of Public Health Forvie Site Robinson Way Cambridge UK; ^2^ Division of Population Health Sciences Royal College of Surgeons in Ireland Beaux Lane House, Lower Mercer Street Dublin 2 Ireland; ^3^ Murdoch Childrens Research Institute, Clinical Epidemiology and Biostatistics Unit The Royal Children's Hospital 50 Flemington Road Melbourne Victoria Australia; ^4^ School of Social and Community Medicine University of Bristol, Oakfield House Bristol BS8 2BN UK; ^5^ School of Life and Medical Sciences, Institute of Clinical Trials and Methodology, Faculty of Population Health Sciences University College London Gower Street London UK

**Keywords:** ALSPAC, FCS, MICE, MNAR, multiple imputations

## Abstract

The not‐at‐random fully conditional specification (NARFCS) procedure provides a flexible means for the imputation of multivariable missing data under missing‐not‐at‐random conditions. Recent work has outlined difficulties with eliciting the sensitivity parameters of the procedure from expert opinion due to their conditional nature. Failure to adequately account for this conditioning will generate imputations that are inconsistent with the assumptions of the user.

In this paper, we clarify the importance of correct conditioning of NARFCS sensitivity parameters and develop procedures to calibrate these sensitivity parameters by relating them to more easily elicited quantities, in particular, the sensitivity parameters from simpler pattern mixture models. Additionally, we consider how to include the missingness indicators as part of the imputation models of NARFCS, recommending including all of them in each model as default practice.

Algorithms are developed to perform the calibration procedure and demonstrated on data from the Avon Longitudinal Study of Parents and Children, as well as with simulation studies.

## 1 INTRODUCTION

Missing data are a common problem in any clinical study. The subsequent loss of information can lead to a reduction in power and biased estimates of clinical effects under many statistical analyses. Methods to deal with the problem of missing data include inverse probability weighting,^1^ Heckman‐type selection modelling,^2^ maximum likelihood estimation,^3^ and imputing the missing data points via principled statistical methods.

Much of the early work on missing data analysis can be traced back to Rubin,^4^, ^5^ which presented a classification of missing‐data mechanisms. Missing data are classed missing at random (MAR) if the probability of missingness depends only on the observed data and not the unobserved data. If missingness does depend on the unobserved data, it is classed as missing not at random (MNAR). When a single variable *Y* is incomplete, MAR assumes that the missing values of *Y* do not differ systematically from the observed values of *Y*, given values of the other variables in the dataset. When multiple variables are incomplete, MAR is a property of the data as a whole^6^: The probability of a particular pattern of missing data depends only on the variables observed in that pattern. Imputation techniques under specific MNAR conditions are becoming increasingly important in practice, as many clinical datasets lack sufficiently detailed covariate information to render the MAR assumption plausible.

By their nature, the MAR and MNAR properties are untestable from the observed data; however, the assumption that data are MAR is historically popular. This is in major part due to the relative simplicity of imputation techniques that assume MAR, which can be performed by joint imputation (all variables at once) via a multivariate normal model^7^ or by fully conditional specification (FCS).^8^ However, such methodology often gives biased results if the data are MNAR. Missing‐not‐at‐random imputation methods, which impose specific departures from MAR, are chiefly handled using joint modelling techniques under pattern mixture models (PMs) and are a well‐documented subject. 9 However, such models come with significant additional complexity compared with those under MAR. As a result, the typical objective of MNAR imputation is to perform a sensitivity analysis (SA) to departures from MAR. This involves contrasting the results of a relevant analysis using MNAR imputed data with the same analysis using MAR imputed data, in order to determine if the analysis under MNAR is associated with a substantial change in conclusions.

Missing not at random SA has previously been performed by comparing a series of competing, fully identified models for the missing data.^10^ More principled SA^11^ is performed by defining a single MNAR model with one of more unidentified parameters, known as sensitivity parameters, allowing the distribution on specific variables to vary between missing and observed data points. Standard SA is then conducted by performing analysis on the imputed data over plausible ranges of these parameters. An alternative technique for SA is a tipping point analysis.^12^ Here, the sensitivity parameter is incrementally increased in magnitude until a qualitative change in inference arising from the analysis occurs, known as the tipping point. One then consults opinion on whether such a departure from MAR is plausible.

Typically, data are imputed under the multiple imputation (MI) technique, which involves imputing multiple versions of the data and then fitting the analysis model on each multiply imputed dataset. The results of each analysis, specifically the estimated effects and standard errors, are then “pooled” using Rubin rules,^5^ which permit analysis that reflects the uncertainty caused by replacing missing values with imputations.

The subject of this paper is the not‐at‐random fully conditional specification (NARFCS) imputation procedure of Leacy.^13^ The procedure is similar to the multiple imputation by chained equations (MICE), or FCS procedure of van Buuren et al,^8^ and shares the same major advantage in that each variable can be modelled by its natural distribution. However, the key difference is that the imputation models include the missingness indicators of the incomplete variables, which allows imputations to be meaningfully different from those one would obtain under FCS and MAR.

There exist methods to apply FCS under MNAR assumptions in van Buuren et al^8^ and Resseguier et al^14^ and the “MI procedure” in the statistical software SAS.^15^ However, these do not include formally defined imputation models. This made it difficult to understand precisely the nature of the sensitivity parameters that were being used. NARFCS constitutes a formalisation of such methods, by defining MNAR imputation models for the chained equations approach. In particular, NARFCS is well suited to imputation of MNAR data with nonmonotone missingness patterns.^16^

This paper has 3 broad aims. The first is to emphasise the exact nature of the sensitivity parameters of the NARFCS procedure, quantities that are conditional on the variables of the data, and how this makes them difficult to elicit (which is obtain plausible values using expert opinion) with current methods. Specifically, we show how misunderstanding the conditional nature of the NARFCS sensitivity parameters can lead to surprising imputations with an example from van Buuren et al.^8^ Secondly, we propose standard procedures as to how to include missingness indicators in the NARFCS imputation models, for which there is currently no guidance. Thirdly, we show a method to choose NARFCS sensitivity parameters by relating them to more elicitable sensitivity parameters from other PMs. This method is illustrated with an example using real data investigating the association between IQ scores in children and young adults and gender, and by simulation.

We describe the main dataset used in the paper in Section 2 and then detail the FCS and NARFCS procedures in Section 3, as well as methods to elicit a sensitivity parameter, and the associated problems in Section 4. We explore the nature of the NARFCS sensitivity parameters in Section 5 and develop methods to choose NARFCS sensitivity parameters in Section 6. A short discussion on the inclusion of missingness indicators, with recommendations, is given in Section 7. The methods of the paper are demonstrated on real data in Section 8 and by simulation in Section 9. Finally, the work of the paper is concluded and possible future work is considered in Section 10.

## 2 DATASETS

The methodology of this paper will be demonstrated on a sample of participants from the Avon Longitudinal Study of Parents and Children (ALSPAC).^17^ Detailed information about ALSPAC is available on the study website (http://www.bristol.ac.uk/alspac), which includes a fully searchable dictionary of available data (http://www.bris.ac.uk/alspac/researchers/data‐access/data‐dictionary). Pregnant women resident in the former Avon Health Authority in south‐west England, having an estimated date of delivery between 1/4/91 and 31/12/92, were invited to take part, resulting in a core enrolled sample of 14 541. Of the 13 988 singletons/twin offspring alive at 1 year, a small number of participants withdrew consent (n = 24) leaving a starting sample of 13 954. Ethical approval for the study was obtained from the ALSPAC Law and Ethics committee and local research ethics committees.

We are interested in 3 variables, gender (0 = Male, 1 = Female), and the IQ of the participants was measured at ages 8 and 15 years, denoted as *I* *Q*_8_ and *I* *Q*_15_, respectively. Data on gender are complete, but data on IQ contain significant missing information, with data for *I* *Q*_8_ missing on 6921 (49.6*%*) individuals and for *I* *Q*_15_ on 9239 (66.2*%*) individuals. Our model of interest examines the association between gender and IQ at age 15:

$$IQ_{15}=\beta_{0}+\beta_{1}Gender+\epsilon .$$

We are primarily interested in illustrating the methods of this paper; however, there is also an interest in exploring how IQ scores can affect missingness patterns between males and females. We analyse the data twice. In the first analysis, missing IQ data are imputed using FCS, assuming the data are MAR. However, we expect the missing data from both IQ variables to systematically differ from the observed. We therefore perform a second analysis when the data are imputed using NARFCS, demonstrating the methods in this paper. In each case, IQ at age 8 is used as an auxiliary variable in the imputation models in order to better inform imputations for IQ at age 15. The objective is to compare the results of both analyses to assess whether the effect of gender on IQ is vulnerable to departures from MAR as a demonstration of the need for imputation under MNAR conditions.

## 3 THE FCS AND NARFCS PROCEDURES

### 3.1 Fully conditional specification

The FCS procedure of van Buuren et al,^8^ sometimes referred to as MICE, is a popular imputation method. Suppose we have *p* random variables ***Y***=(*Y*_1_,…,*Y*_*p*_) containing data on *n* individuals, in which data on some individuals is missing, corresponding binary missingness indicators ***M***=(*M*_1_,…,*M*_*p*_) (with *M*_*j*_=1 if missing and 0 otherwise) and *q* completely observed variables ***X***=(*X*_1_,…,*X*_*q*_). Denote −*j* as the set of all values, less the *j*th element. In FCS, imputations are obtained 1 variable at a time, based on a series of conditional imputation models:

$$P(Y_{j}|X,Y_{-j})\;j=1,\text{⋯},p.$$

These univariate models can be defined to reflect the variable type of each *Y*_*j*_ and are specified as generalised linear models (GLM). We start by imputing in an arbitrary fashion, and then “cycle” through the imputation models. In each cycle, the imputation models for each variable are fitted by regressing on the remaining variables of the model (with their currently imputed values), and imputations are drawn from the resultant posterior predictive distribution of the variable. These cycles are then repeated a number of times until imputations stabilise.

We will impute the ALSPAC data under FCS using the following imputation models:

$$E(IQ_{8}|IQ_{15},Gender)=\alpha_{10}+\alpha_{11}IQ_{15}+\alpha_{12}Gender,$$

(1) $$E(IQ_{15}|IQ_{8},Gender)=\alpha_{20}+\alpha_{21}IQ_{8}+\alpha_{22}Gender.$$

Multiple versions of the data are imputed in this way as per MI, and analysis is performed using Rubin rules. For more detail on FCS and MI, we refer to the tutorial in White et al. 18

### 3.2 The NARFCS Procedure

The NARFCS procedure permits MNAR imputation based on the chained equations methodology, on which FCS is based. The general procedure is as follows (further details can be found in Leacy 13).

1. Define a series of fully conditional distributions for each *Y*_*j*_, involving all remaining *Y*_*j*_, ***X***, and **all** *M*_*j*_ (see Section 7):
  $$P(Y_{j}|X,Y_{-\;j},M_{j},M_{-\;j})\;j=1,\text{⋯},p.$$
  Represent each distribution as a GLM, regressing *Y*_*j*_ on the remaining variables.
2. For each model, choose values for the sensitivity parameters. These are the coefficients of any terms involving *M*_*j*_ in the GLM for *Y*_*j*_ in the above models. These quantities are inestimable from the data, and thus values for them must be set by the user. How to set such values is a key point of the paper with methods developed in Section 6.
3. Then, draw imputations for the data using the chained equations algorithm, where in each cycle, we set
  $$P(Y_{j}|X,Y_{-\;j},M_{j}=1,M_{-\;j})$$
  as the univariate imputation models of the procedure. To fit these models in each cycle, fit the estimable portion of the model (that is all terms not involving *M*_*j*_) via regression. This fits the model
  $$P(Y_{j}|X,Y_{-\;j},M_{j}=0,M_{-\;j}).$$
  Then, add to the fitted coefficients of this model the elicited values for the sensitivity parameters (see below) to obtain the fitted imputation models. Then, draw imputations using this model as one would in typical FCS.

We note that imputing under NARFCS setting all sensitivity parameters to zero is not equivalent to imputing under standard FCS due to the presence of the ***M***_−*j*_ terms in the models. The MNAR imputations created by NARFCS depart from the standard MAR analysis in 2 ways. First, they incorporate terms for the missingness indicator of the variable being imputed, the coefficients of which are sensitivity parameters that cannot be estimated from the data, as they quantify systematic differences between individuals with missing and observed data on that variable. Second, they incorporate the missingness indicators of other incomplete variables as auxiliary predictors. Including the ***M***_−*j*_ terms is discussed in Section 7. Departures from the standard MAR analysis performed by FCS can still be performed by comparing the results of a NARFCS analysis to those of a standard FCS analysis.

We will impute the ALSPAC data under NARFCS using the following linear regression imputation models—as above

$$E(IQ_{8}|IQ_{15},Gender,M)=\beta_{10}+\beta_{11}IQ_{15}+\beta_{12}Gender+\beta_{13}M_{15}+\delta_{8}M_{8},$$

(2) $$E(IQ_{15}|IQ_{8},Gender,M)=\beta_{20}+\beta_{21}IQ_{8}+\beta_{22}Gender+\beta_{23}M_{8}+\delta_{15}M_{15},$$

where ***M***=(*M*_8_,*M*_15_) is the missingness indicators for *I* *Q*_8_ and *I* *Q*_15_, respectively. There are 2 sensitivity parameters for these models, *δ*_15_ and *δ*_8_. To fit the imputation model for *I* *Q*_8_, for example, we fit all the estimable parameters of the model (everything but the *M*_8_ term) and add *δ*_8_ to the fitted intercept. NARFCS can be implemented using modifications to existing software packages for MICE. In this paper, we will perform NARFCS using an extension to the R package “mice,”^8^ detailed in Moreno‐Betancur et al^19^ and currently available on GitHub under the URL “https://github.com/moreno‐betancur/NARFCS.”

## 4 SENSITIVITY PARAMETERS AND ELICITATION

Principled SA requires choosing values, or ranges of values, to vary the sensitivity parameters of the MNAR imputation models. The interpretation of a sensitivity parameter varies based on the imputation model, but is defined as a specified difference in distribution between the missing and observed individuals on a variable. This is typically a difference in mean for continuous variables, or the difference in odds ratio, of observing a positive response for binary variables. Hence, the greater the magnitude of the sensitivity parameter, the more we suppose the missing data depart from MAR.

These chosen values must be plausible for the SA to be relevant. We therefore need to elicit them from expert opinion. This may be achieved either by asking questions to experts or by deriving values based on relevant previous work. Eliciting from an expert directly is notably challenging. An example of the question one might ask is

“Suppose I have two typical groups in the study, individuals in one group gave us information about themselves [on the selected variable], and those in the other did not did not. Suppose the average for the group who did was x, what do you think this value would have been for the group who did not give us this information?”

The key is that the variable, and the information about how the two groups compare are clinically familiar to experts. Such questions usually ask an expert to compare 2 “typical” participants, as this has the effect of eliciting their views independent of all other variables. Experts tend to be more knowledgeable about a variable at the population level rather than on specific groups of people who are matched in certain aspects. Eliciting information on any rarely studied variable or conditional on groups of individuals rarely considered in typical studies is rarely possible, as elicitation requires experts to have intimate knowledge of the subject.

By definition, NARFCS sensitivity parameters are the specified differences between imputed and observed values of a variable, conditional on all remaining variables of the data and their missingness indicators. We therefore refer to them as conditional sensitivity parameters, or CSPs, to distinguish them from sensitivity parameters of almost all other PMs in common use. Most sensitivity parameters are marginal on at least some of the remaining variables^12^ and hence referred to as marginal sensitivity parameters (MSP).

Direct elicitation of a CSP is typically not feasible, as this conditional nature forces one to elicit information about groups of people who are matched in ways that are not commonly analysed, if at all. This makes typical elicitation nearly impossible for NARFCS.

For example, in our NARFCS models for ALSPAC, the CSP *δ*_15_ is the average difference in IQ at age 15 between a responder and a nonresponder, given they are matched on gender, their IQ at age 8, and their missingness pattern for gender and IQ at age 8. While experts may be comfortable conditioning on well‐studied baseline sociodemographic variables such as age and gender, analysis of IQ on groups who happen to have the same IQ score at a previous time is not well studied, nor is conditioning on groups of people with the same patterns of missingness.

Similarly, if a tipping point analysis was conducted with NARFCS as in Leacy,^13^ the tipping point for the CSP does not have a clear clinical interpretation, so it is hard to assert if the tipping point value is even a plausible difference between missing and observed in reality and cast doubt on an analysis under MAR.

## 5 NATURE OF THE NARFCS SENSITIVITY PARAMETERS

Because of the difficulties in eliciting values for a CSP, there is a danger that the user is tempted to elicit values for an MSP instead to put into NARFCS, or that an expert asked for their views will ignore, or misunderstand the conditional nature of the groups they are being asked about. In this section, we show that this can generate imputations inconsistent with the assumptions of the user, as imputed data with differences between the observed and imputed conditional on a variable will display differences marginal on that variable that are a different value.

To explore these issues, we consider the following PM for 2 continuous incomplete variables ***Y***=(*Y*_1_,*Y*_2_) with missingness indicators ***M***=(*M*_1_,*M*_2_). We assume this is the true model for the data.

(3) $$Y|M∼N\left(\left(\begin{array}{c} \mu_{10}+\underbrace{\mu_{11}}M_{1}+\mu_{12}M_{2}\\ \mu_{20}+\mu_{21}M_{1}+\underbrace{\mu_{22}}M_{2} \end{array}\right),\left(\begin{array}{cc} \sigma_{1}^{2} & \rho \sigma_{1}\sigma_{2}\\ \rho \sigma_{1}\sigma_{2} & \sigma_{2}^{2} \end{array}\right)\right).$$

We note that the sensitivity parameters of the PM, *μ*_11_, and *μ*_22_ highlighted by under brackets are MSPs and are the average difference in mean of *Y*_1_ and *Y*_2_, respectively, between an observed and missing individual, marginal on the other *Y*_*j*_ (but conditional on its missingness indicator). We seek to obtain NARFCS imputation models that are an equivalent model for the data as the PM. The CSPs of such a NARFCS model would then be the sensitivity parameters we would need to use when imputing under NARFCS, in order to obtain marginal differences between missing and observed individuals consistent with the MSPs. Clearly, the equivalent NARFCS models for the data are the PMs implied full conditional distributions. These are given as

$$\begin{array}{ccc} & Y_{1}|Y_{2},M_{1},M_{2}∼N\left(\mu_{1|2},\Sigma_{1|2}\right), & \\ & Y_{2}|Y_{1},M_{1},M_{2}∼N\left(\mu_{2|1},\Sigma_{2|1}\right), \end{array}$$

where

(4) $$\mu_{1|2}=E(Y_{1}|Y_{2},M_{1},M_{2})=\mu_{10}+\mu_{11}M_{1}+\mu_{12}M_{2}+\frac{\rho \sigma_{1}}{\sigma_{2}}(Y_{2}-\mu_{20}-\mu_{21}M_{1}-\mu_{22}M_{2})=\left(\mu_{10}-\frac{\rho \sigma_{1}}{\sigma_{2}}\;\mu_{20}\right)+\frac{\rho \sigma_{1}}{\sigma_{2}}Y_{2}+\left(\underbrace{\mu_{11}-\frac{\rho \sigma_{1}}{\sigma_{2}}\;\mu_{21}}\right)M_{1}+\left(\mu_{12}-\frac{\rho \sigma_{1}}{\sigma_{2}}\;\mu_{22}\right)M_{2}$$

(5) $$\mu_{2|1}=E(Y_{2}|Y_{1},M_{1},M_{2})=\mu_{20}+\mu_{21}M_{1}+\mu_{22}M_{2}+\frac{\rho \sigma_{2}}{\sigma_{1}}(Y_{1}-\mu_{10}-\mu_{11}M_{1}-\mu_{12}M_{2})=\left(\mu_{20}-\frac{\rho \sigma_{2}}{\sigma_{1}}\;\mu_{10}\right)+\frac{\rho \sigma_{2}}{\sigma_{1}}\;Y_{1}+\left(\mu_{21}-\frac{\rho \sigma_{2}}{\sigma_{1}}\;\mu_{11}\right)M_{1}+\left(\underbrace{\mu_{22}-\frac{\rho \sigma_{2}}{\sigma_{1}}\;\mu_{12}}\right)M_{2}$$

and

$$\sigma_{1|2}^{2}=(1-\rho^{2})\sigma_{1}^{2},\;\text{and}\;\sigma_{2|1}^{2}=(1-\rho^{2})\sigma_{2}^{2}.$$

We see that the CSP and MSP differ algebraically. For *Y*_1_, the CSP and MSP are given by $\mu_{11}-\frac{\rho \sigma_{1}}{\sigma_{2}}\;\mu_{21}$ and *μ*_11_, respectively (highlighted by under braces). The consequence is that data for *Y*_1_, imputed under this NARFCS model, will show mean differences from the observed marginal on *Y*_2_ $\;\frac{\rho \sigma_{1}}{\sigma_{2}}\;\mu_{21}$ units higher than the posited CSP. In short, if we elicited values for the CSP without correctly conditioning on *Y*_2_, that is, we interpreted the CSP as the MSP, we would usually get imputations that will not show the marginal differences we assumed. It is hence critical to account for the conditional nature of the CSP.

To demonstrate our point, we refer to the following example in van Buuren et al.^8^ It demonstrated an early version of MNAR FCS imputation on the Leiden 85+ Cohort Data. Systolic blood pressure (SBP) was imputed under a normal linear NARFCS model including some baseline covariates (***X***), diastolic blood pressure (DBP), and missingness indicator (*M*_*SBP*_) with sensitivity parameter *δ* as follows. This corresponds to a NARFCS procedure with model.

$$E(SBP|X,DBP,M_{SBP})=X\Psi_{1}+\psi_{1}DBP+\delta M_{SBP}.$$

Diastolic blood pressure was imputed under a regular FCS model and, as such, included only ***X*** and SBP:

$$E(DBP|X,SBP)=X\Psi_{2}+\psi_{2}SBP.$$

It was noted that the mean difference in SBP, marginal on DBP, between imputed and observed was much larger than the specified values of *δ*. This was discussed in van Buuren^20^ and attributed to the cyclic nature of the chained equations algorithm compounding and hence inflating the effect of *δ* over multiple cycles. A remedy was suggested to scale imputed data for SBP by a dampening factor of $\sqrt{1-r^{2}}$, where *r* ^2^ was the proportion of variance explained by the imputation model.

We are now in a position to show, however, that the cause of this apparent inflation is that *δ* is a CSP, which conditions on DBP. Let us return to the NARFCS models of Equations 3, 4, and 5. To obtain NARFCS models that behave like those in this example, we set *μ*_12_ = *μ*_22_ = 0, and the coefficient of *M*_1_ in the NARFCS model for *Y*_2_ to zero, that is, $\mu_{21}-\frac{\rho \sigma_{2}}{\sigma_{1}}\;\mu_{11}=0$. This means the full conditional distributions then become

$$E(Y_{1}|Y_{2},M_{1},M_{2})=(\mu_{10}-\frac{\rho \sigma_{1}}{\sigma_{2}}\mu_{20})+\frac{\rho \sigma_{1}}{\sigma_{2}}Y_{2}+(\mu_{11}-\frac{\rho \sigma_{1}}{\sigma_{2}}\mu_{21})M_{1},$$

$$E(Y_{2}|Y_{1},M_{1},M_{2})=(\mu_{20}-\frac{\rho \sigma_{2}}{\sigma_{1}}\mu_{10})+\frac{\rho \sigma_{2}}{\sigma_{1}}Y_{1}.$$

These correspond to the NARFCS models used in van Buuren et al,^8^ with *Y*_1_=*S* *B* *P*, *Y*_2_=*D* *B* *P*, terms involving ***X*** absorbed into the intercept terms, and $\delta =\mu_{11}-\frac{\rho \sigma_{1}}{\sigma_{2}}\;\mu_{21}$. As *μ*_12_ = 0, from Equation 3, we see that the MSP for *Y*_1_, *μ*_11_ is now the mean difference between observed and imputed marginal on *Y*_2_ and *M*_2_, which is the quantity 20 compared with *δ*. Note that

$$\mu_{21}=\frac{\rho \sigma_{2}}{\sigma_{1}}\;\mu_{11}.$$

Substituting this value into the CSP for *Y*_1_(SBP) then gives

$$\delta =\mu_{11}-\frac{\rho \sigma_{1}}{\sigma_{2}}\;\mu_{21}=\mu_{11}-\rho^{2}\mu_{11}=(1-\rho^{2})\mu_{11}.$$

Hence, differences between observed and imputed data on SBP, conditional and marginal on DBP, naturally differ by a factor of (1−*ρ* ^2^). This explains the discrepancy described in van Buuren,^20^ which was in fact the natural difference between *δ*, the CSP, and the marginal mean of SBP. This was interpreted as incorrect imputations. We can now see the suggested dampening factor is an effect similar to the natural difference between this MSP and the CSP without the square root.

## 6 PERFORMING SA USING NARFCS

### 6.1 Basic strategy

The specification of NARFCS sensitivity parameters must account for their conditional nature, but in doing so, they become almost impossible to elicit from experts with conventional methods (Section 4). However, Section 5 demonstrates that we could relate a CSP to the MSP of another model, which is easier to elicit. In this section, we detail strategies to perform SA based on this idea.

In standard SA, plausible values or ranges for the sensitivity parameters are elicited from experts. When a range is elicited, plausible values are taken over that range. For each value, the data are imputed via MI and analysed via Rubin rules. The results of each analysis are then compared to assess if there is a substantial change in conclusions between them.

As elicitation of a plausible value or range of values of a CSP is particularly difficult, we propose the following basic strategy.

1. Define the NARFCS imputation models for each variable, clearly defining the CSPs we need to determine.
2. Define a set of PMs, one for each incomplete variable, in which the sensitivity parameters are MSPs, marginal on at least some of the variables of the model. These MSPs should have a clear clinical interpretation, and by extension be elicitable from expert opinion. We will call these the MSP models.
3. Elicit plausible values or ranges for the MSPs.
4. For each variable, identify the relationship between the MSP and the CSP, and infer the value/range of the CSP corresponding to the elicited MSP value/range. This is the “calibrated” value/range of the CSP.
5. Perform standard SA using the inferred values/ranges of the CSPs.

Note we do not impute the data under the MSP models. The calibrated value of a CSP is such that if we fit the MSP models to the NARFCS imputed data with this CSP, the imputations show differences from the observed consistent with the elicited MSP. This lets us relate the CSP to other quantities, which are easier to understand and elicit. Performing step 4 is the main challenge. We describe 3 algorithms that can perform step 4 for any choice of NARFCS and MSP models in steps 1 and 2. Algorithm 1 is designed for elicited ranges of MSP values, and algorithms 2 and 3 for elicited single values of the MSP. R codes can be found in the supplementary material, which will perform all 3 of these algorithms.

*The “one‐at‐a‐time” assumption*

We note that calibration of the CSPs is substantially simpler if we can make the assumption that the relationship between the CSP and MSP of one variable is unaffected by the values of the CSPs for any other variable. That is to say, we can calibrate the CSPs “one at a time.” We believe that this assumption is valid in the case of normally distributed missing data with relatively simple choices of MSP models and NARFCS imputation models. In most other cases, such as with binary data and imputation models taking the form of logistic regression models, we believe this assumption is not valid, but there are no formal proofs. In practice, this assumption is easier to verify empirically rather than theoretically, and we will explain how to perform this verification with algorithms 1 and 2 below.

### 6.2 Calibration and SA for elicited ranges of MSPs

It is most common to elicit plausible ranges, rather than single points, for the MSP values. This is probably the easiest way to perform NARFCS SA with calibrated CSPs, as we can perform steps 4 and 5 of the basic strategy using the following algorithm.

Algorithm 1

- Perform steps 1 to 3 of the basic strategy, eliciting for the MSPs ranges of plausible values.
- Define a test range for the CSP of each variable, and take points in regular intervals over each range as test values. See below as to how to do this.
- Create a series of test vectors representing every combination of the test values for the CSPs defined in step (ii).
- For every test vector, fix the random number seed, and impute the data using the NARFCS models in step 1, with CSPs as given in the test vector. Then, fit the MSP models of step 2 via pooled regression to the imputed data, and estimate the parameters corresponding to the MSPs. Use the same random number seed each time.
- With the same multiply imputed dataset in (iv), estimate the “effect of interest” and “corresponding confidence intervals and *P* values.” Do this by fitting to the imputed data your substantive model via Rubin rules or otherwise.
- Check, either graphically or otherwise, that the ranges of the estimated values for the MSPs you obtain include their elicited ranges from step (i).
- If step (vi) is not satisfied for one or more variables, repeat steps (i) to (v) with wider test ranges of the relevant CSPs, or center the ranges at a different value, until step (vi) of this algorithm is satisfied.
- To perform SA, simply graph (or tabulate) the estimated values for the effect of substantive interest against the estimated MSP values you obtained, and mark the elicited ranges.

*Comments on algorithm* 1

The above algorithm allows one to assess the evolution of your effect of interest, and its *P* value for NARFCS imputed data showing differences between imputed and observed individuals consistent with elicited ranges for the MSPs.

However, the algorithm can be intensive computationally. For example, with 2 CSPs each taking 10 test values, the algorithm must run MI 100 times. For this reason, we suggest at step (ii) to pick fairly wide ranges for the CSP. This width will depend on the scale of the variable, but we recommend centring it on the elicited range of the MSP and taking test values at fairly large intervals so you obtain no more than 10. Ideally, you would want at least a few test vectors to fall within your elicited MSP ranges. If the widths between your test values are too wide for this, you can rerun the algorithm using the results from the first run to obtain more test vectors that fall in the range you are interested in.

Using graphical methods to analyse the results becomes difficult when are more than 2 MSPs. However if the one‐at‐a‐time assumption is valid, SA can simply plot the estimated MSPs against the effect of interest for each variable independently, offering easier to read results. We can validate this assumption by tabulating the test points (CSPs) and estimated MSPs of algorithm 1, and seeing if the difference between the CSPs and MSPs of one variable noticeably change when the CSPs of other variables are changed.

### 6.3 Tipping point analysis

A tipping point analysis using NARFCS was conducted in Leacy.^13^ By regularly increasing the magnitude of your CSPs in regular increments, each time imputing the data and noting a *P* value or confidence interval for Rubin pooled regression on the effect of interest, one can identify values for the CSPs whereby a qualitative change in conclusions about that effect occurs. These CSP values are the “tipping points” of the analysis. This can be performed automatically with algorithm 1 above, through sensible choices for the test ranges of each CSP and simply observing the *P* values of the significance tests you obtain.

Performed this way we can express the tipping points as MSP values, which are easier to understand and as such allow us to assert whether the tipping points represent plausible departures from MAR. This can either be left up to the reader, or an elicitation exercise can be undertaken to investigate the extent to which expert opinion finds the tipping points plausible. Note that these experts, and indeed the investigators obtaining their opinions, should not know what the tipping points are in advance; else, we could easily introduce bias in the way questions are formed and the opinions they give.

### 6.4 Calibration for specific elicited MSP values

Algorithm 1 is fairly comprehensive for elicited ranges of MSPs; however, in some cases, it is of interest to perform SA at specific values of a sensitivity parameter. This can occur when the sensitivity parameters are derived based on a calculation using data from previous works, as we will do in Section 8 of this paper. Other times, elicitation exercises obtain a set of plausible values from experts, rather than a plausible range, such as in Wallace et al.^21^, Table S8 Even when a plausible range for an MSP is elicited, interest may lie specifically at the lower and upper limits of the range.

For this, a means is needed to identify the calibrated CSP at particular values of an MSP, for which algorithm 1 is not suited. Interpolation of the results of algorithm 1 between the 2 CSPs that have corresponding MSPs closest to the elicited value is an option but may be imprecise if the distance between them is large, or if the relationship between the MSP and CSP is nonlinear. In this section, we describe 2 algorithms designed for the calibration of elicited points of MSPs.

Algorithm 2 all CSPs at once

A p‐dimensional search, identifying the calibrated CSPs for all the elicited MSPs at once, can be performed by repeated application of a similar algorithm to algorithm 1.

- Perform steps 1 to 3 of the basic strategy.
- Perform steps (ii) to (iv) of algorithm 1.
- Choose a tolerance for each MSP, and identify the test vector (if one is identified) whose corresponding MSP estimates lie within the specified tolerances. Take the test vector as the calibrated CSPs, and observe the estimated MSP, effect of interest, and test of its significance as your SA.
- If no test vector is valid, repeat steps (ii) and (iii) with different test ranges for the CSP or test values taken in closer intervals. Alternatively increase your tolerances for the estimated MSPs. See below as to how to repeat steps (ii) and (iii).

It is very unlikely that a single run of algorithm 2 will yield acceptably accurate calibrated CSPs. The lower your tolerances for step (iii) are, the more accurate your CSP calibration. However, this requires one to take smaller intervals between test points in step (ii) to locate a valid test vector. Typically, the distances between test points need to be the same values as your tolerances to identify a valid test vector, which with most accepted levels of tolerance will dramatically increase computational time if done with 1 run of the algorithm.

We instead propose first running the algorithm with wide test ranges with large intervals between test points and tolerances. Using the results of this algorithm, identify the CSPs whose estimated MSPs come closest to the elicited values. Then, rerun the algorithm with these as the limits of the test ranges for the CSPs and take smaller intervals between test values. Continue this until you obtain a test vector within appropriate tolerances. We note that it may be possible to find multiple CSPs that display imputed data with the correct values for the MSPs.

Algorithm 3 calibrating point CSPs one at a time

Since algorithm 2 amounts to repeated application of algorithm 1, computation time may be lengthy. However, if after the first application it becomes clear that the one at a time assumption is valid (as explained below algorithm 1), we can use the following more efficient algorithm to calibrate the CSPs for specific elicited values of the MSPs one variable at a time.

- Perform steps 1 to 3 of the basic strategy.
- Decide on an initial range to vary the CSP of the chosen variable. This range should be wide and typically include the elicited value of the corresponding MSP. Take points in regular intervals over these ranges. We refer to these as “test values.”
- Fix the CSPs for the remaining variables at sensible values, such as the elicited value of the corresponding MSP.
- Fix a random number seed, then perform NARFCS under the models in step 1 with a test value of the CSP from step (ii). Use this same random seed every time you return to this step.
- Using the multiply imputed data resulting from step (iv), fit the relevant MSP model of step using Rubin rules, and estimate the parameter that corresponds to the MSP.
- Repeat steps (iv) and (v) for every test value of the CSP over the interval in step (ii).
- Determine the 2 test values for which the estimated MSPs of step (v) are the closest to the elicited value (lesser and greater than). Then, return to step (ii) with these test values as the lower and upper bounds of the initial range and repeat.
- Repeat steps (ii) to (vii) until convergence of the 2 estimated MSPs in step (vii) to within a specified tolerance. Then, take the test value whose estimated MSP is closest to the elicited value as the calibrated CSP.

Repeat this algorithm for each CSP in turn. Then, perform SA using these calibrated CSPs when you impute. We again note that algorithms 1, 2, 3 can be performed using R code that we have included as supplementary material.

## 7 INCLUSION OF MISSINGNESS INDICATORS IN NARFCS MODELS

Besides the elicitation of sensitivity parameter values, the other outstanding issue with NARFCS pertains to how one should include the missingness indicators in the univariate NARFCS imputation models. Previous works (van Buuren et al^8^ and Resseguier et al^14^) are examples of NARFCS where the imputation models for each variable include only its own missingness indicator. There is currently little to no guidance as to which missingness indicators to include in each of the models, or how this might affect the accuracy of imputations.

We recommend that the NARFCS imputation model for each incomplete variable *Y*_*j*_ should include the missingness indicator for the incomplete variable *M*_*j*_ (the effect of which is the CSP), as well as the missingness indicator of any other incomplete variable *M*_*k*_ whose effect in the imputation model can be estimated from the observed data.

This reasoning is to ensure that all (or as much as possible) of the correlation between variables is captured as part of the imputation model. As we are imputing based on a series of conditional models, there is a realistic possibility that a variable *Y*_*i*_ will become correlated with some other *M*_*j*_, once it has been conditioned on the corresponding *Y*_*j*_. In other words, it is plausible that the true full conditional distribution of a variable will depend on the missingness indicators of other variables. Our interest is not in determining the association between the missingness indicators and substantive variables but in capturing any correlations between them. As such, their omission in the NARFCS models may lead to a loss in information from which to inform imputations and possible bias. If the procedure models an unnecessary conditional dependence relationship between the ***Y*** and ***M***, it should not have a negative impact on imputations.

When no pair of variables have a monotone missing data pattern, all of the associations between missingness indicators and substantive variables (that are not specified as sensitivity parameters) can be estimated from the observed data. This is since there exist observed data points in every missingness pattern for the data. In this case, every NARFCS imputation model should include every missingness indicator. When this is not the case, such as in longitudinal or single drop out data, some of these associations can no longer be estimated, and we prefer to remove such associations from the NARFCS models.

The NARFCS procedure in R^19^ will automatically remove variables (not specified as sensitivity parameters) from the imputation models that cannot be estimated. Hence, when using the software, the user should include all the missingness indicators by default and note any that the procedure removes. We are currently investigating how best to include missingness indicators in the longitudinal data setting.

## 8 EXAMPLE: ALSPAC DATASET

### 8.1 Overview

We now conduct our analysis of the ALSPAC dataset that was introduced in Section 2. Recall that we will perform 2 analyses, the first using FCS and the second using NARFCS. The imputation models for these analyses can be found in Section 3 as Equations 1 and 2, respectively. For each, we will fit the analysis model using Rubin rules and model the association between Gender on *I* *Q*_15_ to assess if this effect is vulnerable to departures from MAR.

We expect the data on these 3 variables to be MNAR, and hence that NARFCS imputation may be more appropriate. We then need to choose values for the 2 sensitivity parameters of the model *δ*_8_ and *δ*_15_. Hence, we apply the methodology of Section 6. We calibrate the CSPs of both IQ variables so that imputations are in line with MSPs, *μ*_8_ and *μ*_15_, interpreted as the mean difference in IQ between observed and imputed, marginal on all other variables. These come from the 2 MSP models

$$E(IQ_{8}|M_{8})=\mu_{8,0}+\mu_{8}M_{8},$$

(6) $$E(IQ_{15}|M_{15})=\mu_{15,0}+\mu_{15}M_{15}.$$

We choose a value for *μ*_15_ as −7.3; that is, we expect missing *I* *Q*_15_ to be 7.3 points lower on average. This is a derived calculation based on the work of Cornish et al.^22^, p. 942 The mean of IQ in the observed data (31%) of the data was 94.4, and after imputation, the mean of the total IQ was 89.4. The latter was a weighted average of observed IQ, and imputed IQ, accounting for 69% of the data, imputed using MAR‐based methods but accounting for the data being MNAR by including linked educational attainment data as an auxiliary variable, which strongly predicted missingness in IQ. We then calculated that the mean of the imputed data, assumed to be MNAR, was approximately 87.1. The difference between observed and imputed is therefore 7.3. For simplicity, we also set the value of *μ*_8_ to −7.3.

We now calibrate the CSPs as detailed in Section 6 using algorithm 3, having predetermined that the one‐at‐a‐time assumption is valid. We set an initial range for both *δ*_8_ and *δ*_15_ as [−20,0], taking points 1 unit apart. For step (iii), we set the CSP for the other IQ variable to −10 and set the tolerance as 0.001. In this algorithm, NARFCS was performed with 10 cycles and 10 multiply imputed datasets. This gave us calibrated CSP values *δ*_8_≈−0.556 and *δ*_15_≈−1.780. In fact, plotting the MSP and CSP values shows that their relationship is linear, and approximately *δ*_15_ ≈ *μ*_15_+5.520 and *δ*_8_ ≈ *μ*_8_+6.744.

### 8.2 Results

We now impute the data under both FCS and NARFCS procedures. In both procedures, we generate 10 multiply imputed data sets, with 10 cycles, and fit 2 models to the data under Rubin rules. Firstly, we fit the analysis model as shown in Section 2, for both the FCS and NARFCS imputed data, and observe the coefficient of gender. Secondly, we fit the PMs of Equation 6 from which the MSPs were derived, to check if the NARFCS imputed data show the correct marginal differences from the observed. This gave the following results.

Table 1 shows that gender has a weakly significant association with the 15 year IQ score of individuals, when imputing under MAR. Though the effect size (−0.87) appears quite small, it is based on analysis of a large sample size of the dataset. The inference is that females in the dataset have a lower IQ than males by an average of 0.87 points, though the most likely explanation for this is bias due to missing data not being imputed under the correct assumption. We do not expect missing data on IQ to be MAR, and this analysis is intended as a benchmark to assess departures form MAR when imputing under NARFCS.

**Table 1:** Analysis of ALSPAC Dataset By Rubin's Rules Under FCS and NARFCS

Method	Model Fitted	Parameters	est	95% CI	
FCS	Analysis Model	Intercept	91.64	90.10	92.12
		Gender (1=Female)	‐0.87	‐1.73	‐0.02
NARFCS	Analysis Model	Intercept	87.42	86.52	88.33
		Gender (1=Female)	‐0.61	‐1.21	‐0.01
NARFCS	PM for *I* *Q*_8_	Intercept	104.16	103.77	104.54
		*M*_8_	‐7.30	‐9.19	‐5.41
NARFCS	PM for *I* *Q*_15_	Intercept	91.96	91.59	92.34
		*M*_15_	‐7.30	‐8.55	‐6.05

Analysis under the NARFCS imputed data shows that the magnitude of the association between IQ and gender has been cut by close to a third. Furthermore, the intercept term, that is, the mean of male IQ, has also been reduced by 4 points. In both analysis, the 95% CI implies that the association between gender and IQ is weak, particularly for the analysis under NARFCS.

Another possible explanation for the observed association under the MAR analysis could be that males with lower IQ scores are less likely to participate than females with low IQ scores. To test this, we could consider a future analysis that imputes under NARFCS with different sensitivity parameters for each gender, in order to reflect that missing males and females may depart from the MAR assumption differently.

The final 2 models indicate that the imputed data are consistent with the elicited values of the MSPs, inferring a successful calibration of the CSPs. Note the same random seed was used both for the calibration procedure and imputation for the substantive analysis. In practice, this is not necessary, and one would expect some random variation when the random seeds differ.

### 8.3 Tipping point analysis

We can combine our calibration procedure with a tipping point analysis using algorithm 1. Similar exercises, using other imputation methods, can be seen in Moreno‐Betancur et al. 23, 24

We will perform an analysis using NARFCS in the same way as in the results section for pairs of values of *δ*_8_ and *δ*_15_. We will set values for *δ*_8_ at −0, −0.5, −1.5, −2.5, and −3.5 units and values for *δ*_15_ at −1, −2, −3, −4, and −5 units. For all 25 subsequent pairs, we report the association between gender and *I* *Q*_15_ as in section 8.2. We also present a contour plot of the (pooled) *P* value, testing the statistical significance of this association for each pair. This gave the following results.

**Figure 1:**
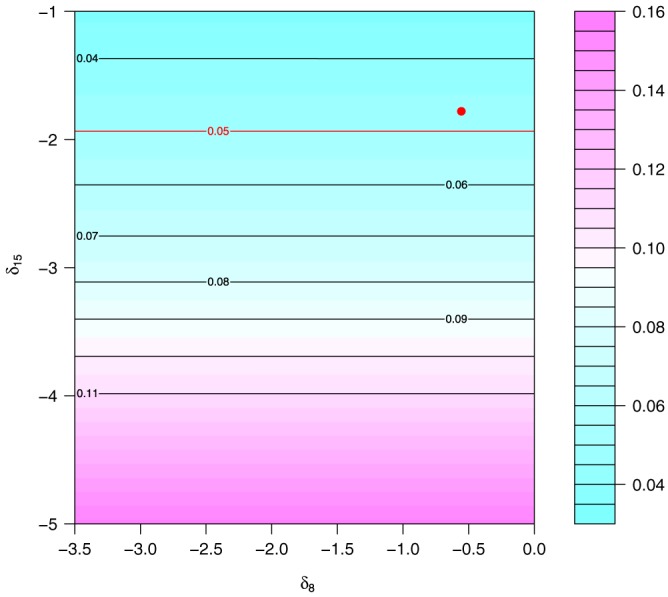
Contour plot of significance tests (P value) for the pooled effect of gender on I Q_15_. The red dot indicates the calibrated values of the conditional sensitivity parameters used in section 8.2, and the red contour (.05) indicates the point where values for δ_15_ below the contour will yield an analysis with p<.05 [Colour figure can be viewed at http://wileyonlinelibrary.com]

Both Table 2 and Figure 1 suggest that the association between gender and *I* *Q*_15_ is very insensitive to changes in the departure from MAR in *I* *Q*_8_, in both the magnitude, and its significance. This is expected as our analysis model does not include *I* *Q*_8_. The association is however sensitive to changes in the departure from MAR in *I* *Q*_15_. There is a notable reduction in the effect size of gender as *δ*_15_ is increased, with the effect size almost halved compared with the MAR analysis when *δ*_15_=−5. If we base our tipping point on the 95% significance test, then conclusions are changed whenever *δ*_15_ ≤ −2, and hence, −2 is the tipping point. The effect size at this tipping point (−0.599) has been reduced by around 31% compared with the same effect under the MAR analysis.

**Table 2:** Results of tipping point analysis

	*δ*_15_					
		‐1.0	‐2.0	‐3.0	‐4.0	‐5.0
***δ*_8_**	**0.0**	−0.652∗	−0.599	−0.546	−0.493	−0.440
	**‐0.5**	−0.652∗	−0.599	−0.546	−0.493	−0.440
	**‐1.5**	−0.652∗	−0.599	−0.546	−0.493	−0.440
	**‐2.5**	−0.652∗	−0.599	−0.546	−0.493	−0.440
	**‐3.5**	−0.652∗	−0.599	−0.546	−0.493	−0.440

∗ An effect where *p*<.05.

A tipping point of −2 corresponds to a value in terms of *μ*_15_ of approximately −7.52; hence, we conclude that the association between gender and IQ score at age 15 is vulnerable to departures from MAR, in particular when missing individuals have a (marginal) average IQ score approaching 7.5 points less than observed individuals. Given how close this tipping point is to the derived value of *μ*_15_, we would suggest such a departure is plausible in reality and that the analysis using MAR imputation may be misleading.

## 9 SIMULATION STUDY

As a final exercise, we will perform a simulation study. We will assess the accuracy of the CSP calibration procedure of Section 6 and look to demonstrate how not correctly conditioning the CSP may also lead to bias.

We simulate 1000 datasets with n = 1000 data points under the bivariate normal PM of Equation 3 in Section 5. The missingness indicators *M*_1_ and *M*_2_ are independently generated from Bernoulli distributions with probabilities .3 and .25, respectively, and we set

$$\mu_{01}=10,\;\mu_{11}=-2,\;\mu_{12}=1,\;\sigma_{1}=2,\;\mu_{02}=20,\;\mu_{21}=1,\;\mu_{22}=-3,\;\sigma_{2}=4,\;\rho =0.6.$$

We then set the relevant data points to missing based on the values of the *M*_*i*_. Recall that the equivalent NARFCS models, given as the full conditional models of Equation 3, were derived in Equations 4 and 5. As such, the true MSP values of the PM are *μ*_11_ = −2 and *μ*_22_ = −3, and the true CSP values are $-2-\frac{\rho \sigma_{1}}{\sigma_{2}}=-2-0.3=-2.3$ and $-3-\frac{\rho \sigma_{2}}{\sigma_{1}}=-3-1.2=-4.2$.

We perform 3 analyses on the simulated data, in each we will impute under a variation of the NARFCS models of Equation 3 with *m*=10 imputed datasets.

- The true NARFCS models: We impute using exactly the NARFCS models of (3), including the true values of the CSPs. This is the “perfect” situation in theory, although not practically possible. It presents the benchmark performance for the remaining simulations.
- Input the MSPs *μ*_11_ and *μ*_22_ directly into NARFCS as though they were CSPs. This is one of the likely errors one may commit in practice.
- We use the correct NARFCS models, set the MSPs at their true value, and calibrate CSPs using algorithm 3 of Section 6. This is the strategy we recommend in practice.

To assess the accuracy of each procedure, we will measure the bias, empirical standard error, and 95% coverage of 2 target parameters, the overall means *E*(*Y*_1_) and *E*(*Y*_2_), which have true values:

$$E(Y_{1})=10-2\;*\;0.3+0.25=9.65,\;\;E(Y_{2})=20+0.3-3\;*\;0.25=19.55.$$

This is achieved by fitting linear models to the imputed data for *Y*_1_ and *Y*_2_ involving only an intercept. We additionally include the maximum Monte Carlo error for each of these estimates. This gave the following results.

Table 3 shows that each analysis displays low Monte Carlo error, as we would expect with 1000 simulations. Also as expected, the first simulation obtains unbiased estimates of the marginal means that are well within the empirical standard error as well as close to nominal coverage.

**Table 3:** Results of simulation study

Method	Parameter	True Value	Bias	Empirical SE	Coverage, 95*%*
True	*E*(*Y*_1_)	9.65	0.000	0.080	96.1
	*E*(*Y*_2_)	19.55	0.000	0.151	95.0
Insert MSPs	*E*(*Y*_1_)	9.65	0.090	0.078	79.3
	*E*(*Y*_2_)	19.55	0.301	0.147	49.3
Calibrated CSPs	*E*(*Y*_1_)	9.65	0.000	0.083	95.0
	*E*(*Y*_2_)	19.55	0.002	0.153	95.1
Max MC error			0.005	0.003	1.581

Abbreviations: CSP, conditional sensitivity parameter; MC, Monte Carlo; MSP, marginal sensitivity parameter.

Inputting the MSP values directly into NARFCS causes significant bias and drop in coverage for both target parameters. Noting that the performance is much worse for *E*(*Y*_2_), where the difference between the true CSP and MSP is larger, suggests that the bias is worsened the more the CSP value is misspecified.

Estimation of the true CSP value via the calibration procedure has performed well against the benchmark. The estimates show little evidence of bias, and coverage remains close to its nominal value. A minor increase in empirical standard error compared to the benchmark “true” simulation implies some loss in precision as expected, which could be alleviated by increasing the number of cycles the NARFCS procedure uses during calibration.

## 10 DISCUSSION

The overall goal of this paper is to allow NARFCS to be correctly implemented by the user with ease, in order to aid in the wider adoption of NARFCS as a practical imputation method for multivariate missing data. Elicitation of the CSP values represented a significant stumbling block to its wider use; however, the work of this paper addresses this issue without too much additional complexity. In fact, the work of this paper permits the use of NARFCS to assess the departure from MAR based on sensitivity parameters with any conditional nature.

An immediate future aim is to adapt the R code found in the appendix, which performs the calibration algorithms, into formal functions as part of an R package. This will make choosing CSP values for NARFCS as simple as defining the MSP models with the desired MSPs, eliciting values for these MSPs, and determining the corresponding CSP values by using the functions. There is also future interest in combining the work on NARFCS and this paper with the work in Yuan^15^ possibly as part of the MI procedure to allow for better use of NARFCS with SAS.

Future work could determine the situations in which the one‐at‐a‐time assumption, required for algorithm 3 and defined in Section 6, is appropriate theoretically, without the need to validate it empirically with algorithms 1 or 2. With this assumption, we can approach calibrating *p* CSPs as a series of *p*‐independent problems. This means we can calibrate large numbers of CSPs without any additional complexity. Furthermore, in algorithm 1, we need only to graph the parameter of interest against each MSP individually.

Practical use of these methods in real life applications should also be a main area for future work. In particular, we look to apply these methods to cases involving binary, and a mixture of binary and continuous data. One major area for which NARFCS may be of use is longitudinal PMs. Such models were explored in Ratitch et al,^16^ in which imputations for a variable at a particular time point were allowed to depend on imputations of the variable at previous time points. Such models are partially conditional, and Ratitch et al^16^ suggested the need for methods to impute under such models, for which NARFCS, paired with the calibration method, may be well suited.

As a final note, we can generalise the methods found in Section 6. So far, it has been used to establish a relationship between an MSP and CSP or to plot a valid range of MSP values against a quantity of interest. Instead, one could estimate the relationship between any summary statistic, such as a known marginal mean from another source, and a quantity of interest as follows.

- Impute *Y* ^mis^|*Y* ^obs^ using the NARFCS procedure over a range for the CSP.
- Estimate the parameter of interest $\;\hat{\beta }(Y^{\text{total}})$ via Rubin rules on the imputed datasets.
- Estimate some interpretable summary from the imputed datasets $Ŝ(Y^{\text{total}})$. Ensure the ensuing range is of practical interest to the user.
- Plot $\;\hat{\beta }$ vs Ŝ.

This represents a generalisation of algorithm 3, using an interpretable summary in place of the MSP, and is possibly a powerful tool for sensitivity analyses.

## Supporting information

Supporting info item
