# Supplementary material for: On the use of the not‐at‐random fully conditional specification (NARFCS) procedure in practice
Source: Stat Med. 2018 Apr 2;37(15):2338–53. doi: 10.1002/sim.7643 (PMC6001532; doi:10.1002/sim.7643)
Supplement: Supplementary file 1 — Supporting info item [file SIM-37-2338-s001.docx]

#Daniel Tompsett January 2018

#Install the required software

library(devtools)

install_github("moreno-betancur/mice",

ref="f0e838f5a7fdfcfb9bfa27207e903d13d79cefaa")

library(foreign)

library(mice)

#To use NARFCS with the "mice" function refer to instructions in

#https://github.com/moreno-betancur/NARFCS.

#

#PREAMBLE:

#Before each algorithm, create the arguments for use in the function "mice"

#Except for the argument parmSens, this will change as part of the algorithm,

# data=The data to impute

# m=Number of Multiply imputed datasets

# maxit=Number of Cycles

# method=Imputation Methods

# predictorMatrix=Predictor Matrix, involving all estimable terms

# predictorSens=Predictor Matrix for sensitivity parameters

# seed=Random seed

# parmSens= These contain CSPs, and are left UNSPECIFIED here

#

# The arguments of the calibration functions are provided using a

# structure following that of the 'parmSens' argument, which are used

# in the procedure to fix a range of possible values for the entries

# of 'parmSens', that is the CSPs. Be sure to understand the structure

# of this argument before attempting calibration

#

# The algorithms here only support MSP models with 1 sensitivity

# parameter per model. The code would need to

# be modified for models with multiple MSPs at once.

############################################################################

#######################Algorithm 1: Elicited ranges of MSPs#################

############################################################################

#Input:

# MSPmodels=List of MSP models in form list(,...)

# Written in the form X~Y (no quotations)

# THE FIRST ENTRY AFTER ~ MUST BE THAT FOR WHICH THE

# THE COEFFICIENT IS THE MSP.

#

# MSPfamily=Vector of MSPmodels regression families in form c("",...)

# arguments passed to family argument of glm.mids

# "gaussian" for normal linear regression

# "biomial" for logistic regression

#

# Substantive=Your substantive model of interest

# Written in the form X~Y (no quotations)

# THE FIRST VARIABLE ENTRY AFTER ~ MUST BE THAT FOR WHICH

# THE COEFFICIENT IS THE MAIN TARGET PARAMETER.

#

# Subfamily= Substantive model's regression family, written as c("")

#

# Intercept=If effect of interest is the intercept, set to TRUE

# Set as FALSE by default

#

# l=Vector of lower bounds of test ranges for the CSPs

# u=Vector of upper bounds of test ranges for the CSPs

# step= Vector of intervals in which test points are taken

#

# parms=List with the same structure as one would use if one were

# specifying the 'parmSens' argument, with ALL entries set as NA

#

# NOTE:

# The arguments parms, l, u and step fill in the entries of 'parmSens'

# with values over fixed ranges of the CSPs: parms gives the structure

# of 'parmSens',and l, u and step fill in the entries of 'parmSens'

# with values between l and u in intervals of length step. Entries

# in 'parmSens' corresponding to complete values are set to "".

#

# Hence l, u and step must have an entry for each

# element of parmSens in the order in which they appear. Set

# l,u and step for entries corresponding to complete variables to NA.

calibrationr.fn<-function(MSPmodels,MSPfamily,Substantive,Subfamily,Intercept=FALSE,l,u,step,parms){

MSP<-as.list(NULL)

substantive<-as.list(NULL)

range<-as.list(NULL)

for(k in 1:length(l)){

if (is.na(l[k])==TRUE){

range[[k]]<-list("")

}

else{

range[[k]]<-seq(l[k],u[k],step[k])

}

}

points<-expand.grid(range)

for(j in 1:nrow(points)){

parmsnew<-relist(as.matrix(points[j,]),parms)

parmSens<-parmsnew

set.seed(seed)

narfcs<-mice(data=data,m=m,maxit=maxit,method=method,

predictorMatrix=predictorMatrix,predictorSens=predictorSens,

parmSens=parmSens,print=F)

MSPlist<-as.vector(NULL)

for(n in 1:length(MSPmodels)){

MSPlm<-glm.mids(MSPmodels[[n]],narfcs,family=get(MSPfamily[n]))

MSPlist[n]<-pool(MSPlm)$qbar[2]

}

MSP[[j]]<-MSPlist

lms<-glm.mids(Substantive,narfcs,family=get(Subfamily))

if (Intercept==TRUE){

substantive[[j]]<-summary(pool(lms))[1,c(1,5:7)]}

else{substantive[[j]]<-summary(pool(lms))[2,c(1,5:7)]

}

}

Msps=do.call(rbind,MSP)

colnames(Msps)<-rep("c",ncol(Msps))

for(i in 1:ncol(Msps)){

colnames(Msps)[i]<-paste("MSP",i)

}

Csps<-points

colnames(Csps)<-rep("c",ncol(Csps))

for(i in 1:ncol(Csps)){

colnames(Csps)[i]<-paste("CSP",i)

}

analysis=do.call(rbind,substantive)

results=cbind(Msps,analysis)

return(list(Csps=Csps,Msps=Msps,

analysis=analysis,

results=results)

)

}

#OUTPUT:

# CSPs=Full list of tested sets of CSPs

# Given in the same order as they appear in parmSens

# Msps=Full list of estimated MSPs

# Given in the same order they appear in MSPmodels

# analysis=List of substantive effects of interest

# With associated p value and 95% confidence interval

# Results=The Msps and substantive analysis appended together

############################################################################

###############Algorithm 2: Elicited Values All Variables at Once###########

############################################################################

#Input:

# MSPmodels, MSPfamily, l, u, step and parms, All same as in algorithm 1

# Two new arguments

# elicited=Vector of elicited values of the MSPs

# tol=Vector of tolerances for each MSP for the algorithm

# elicited and tol are written as c(,...)in same order as MSPmodels

calibrationAAO.fn<-function(MSPmodels,MSPfamily,elicited,l,u,step,tol,parms){

MSP<-as.list(NULL)

range<-as.list(NULL)

for(k in 1:length(l)){

if (is.na(l[k])==TRUE){

range[[k]]<-list("")

}

else{

range[[k]]<-seq(l[k],u[k],step[k])

}

}

points<-expand.grid(range)

for(j in 1:nrow(points)){

parmsnew<-relist(as.matrix(points[j,]),parms)

parmSens<-parmsnew

set.seed(seed)

narfcs<-mice(data=data,m=m,maxit=maxit,method=method,

predictorMatrix=predictorMatrix,predictorSens=predictorSens,

parmSens=parmSens,print=F)

MSPlist<-as.vector(NULL)

for(n in 1:length(MSPmodels)){

MSPlm<-glm.mids(MSPmodels[[n]],narfcs,family=MSPfamily[n])

MSPlist[n]<-pool(MSPlm)$qbar[2]

}

MSP[[j]]<-MSPlist

}

dif<-as.vector(NULL)

for(p in 1:nrow(points)){

dif[[p]]<-tol-abs(MSP[[p]]-elicited)

}

Msps=do.call(rbind,MSP)

colnames(Msps)<-rep("c",ncol(Msps))

for(i in 1:ncol(Msps)){

colnames(Msps)[i]<-paste("MSP",i)

}

colnames(points)<-rep("c",ncol(points))

for(i in 1:ncol(points)){

colnames(points)[i]<-paste("CSP",i)

}

Csps<-points

con<-lapply(dif,function(x)all(x>0))

MSPcal<-MSP[c(which(con==TRUE))]

calibratedMSP=do.call(rbind,MSPcal)

colnames(calibratedMSP)<-rep("c",ncol(calibratedMSP))

for(i in 1:ncol(calibratedMSP)){

colnames(calibratedMSP)[i]<-paste("MSP",i)

}

CSPcal<-points[c(which(con==TRUE)),]

colnames(CSPcal)<-rep("c",ncol(CSPcal))

for(i in 1:ncol(CSPcal)){

colnames(CSPcal)[i]<-paste("CSP",i)

}

return(list(calibratedMSP=calibratedMSP,calibratedCSP=CSPcal,

Csps=Csps,Msps=Msps))

}

#OUTPUT:

# calibratedCSP= Sets of calibrated CSPs which satisfy the tolerances

# calibratedMsp= Estimated MSPs of caliibratedCSP

# Csps=Full list of tested sets of CSPs

# Msps=Full list of estimated MSPs

############################################################################

#######################Algorithm 3: One variable at a time##################

############################################################################

#INPUT:

# MSPmodels, MSPfamily, l, u, step, elicited and tol

# have same meaning as in algorithm 1. Since there is only 1 variable

# no need to write them in as list(,...) or c(,...).

#

# parms=List with the same structure as one would use if one were

# specifying the 'parmSens' argument.

#

# SPECIFIED DIFFERENTLY TO ALGORITHMS 1 and 2

# write as one would write 'parmSens', with the CSP you wish

# to vary set to NA. Set other CSPs to their chosen fixed

# values, and complete variables to "".

calibrationOAAT.fn<-function(MSPmodel,MSPfamily,elicited,l,u,step,tol,parms){

MSPr<-as.list(NULL)

CSPr<-as.list(NULL)

range<-seq(l,u,step)

i<-1

test1<-100

test2<-0

while(abs(test1-test2)>tol){

dif<-as.vector(NULL)

for(j in 1:length(range)){

parmSens<-parms

for(l in 1:length(parmSens)){

parmSens[[l]][is.na(parmSens[[l]])]<-range[j]

}

set.seed(seed)

narfcs<-mice(data=data,m=m,maxit=maxit,method=method,

predictorMatrix=predictorMatrix,predictorSens=predictorSens,

parmSens=parmSens,print=F)

MSPlm<-glm.mids(MSPmodels,narfcs,family=MSPfamily)

dif[j]<-pool(MSPlm)$qbar[2]

}

diff<-dif-elicited

indexp<-which(diff==min(diff[which(diff>=0)]))

indexn<-which(diff==max(diff[which(diff<=0)]))

MSPr[[i]]<-c(dif[indexn],dif[indexp])

CSPr[[i]]<-c(range[indexn],range[indexp])

test1<-dif[indexn]

test2<-dif[indexp]

lower<-min(range[indexn],range[indexp])

upper<-max(range[indexn],range[indexp])

range<-seq(lower,upper,(step/10^i))

i<-i+1

}

return(list=c(Msp=MSPr[i-1],Csp=CSPr[i-1]))

}

#Output:

# Csp=The two closest calibrated values of the CSP within tolerance

# Msp=The estimated MSP values for Csp

###############################################################################

##############################Example##########################################

###############################################################################

#GENERATE EXAMPLE DATASET:

# 2 continuous variables with missing data, Y1 and Y2

# Corresponding missingness indicators M2 and M2

# Complete continuous variable X

set.seed(45678)

c1<-c(20,10,8)

c2<-c(10,15,11)

c3<-c(8,11,14)

sigma<-rbind(c1,c2,c3)

M1<-rbinom(1000,1,0.5)

M2<-rbinom(1000,1,0.5)

mu1=50-(1*M1+2*M2)

mu2=50-(4*M1+5*M2)

mu3=rep(50,1000)

library(MASS)

data<-matrix(,nrow=1000,ncol=3)

for(j in 1:1000){

data[j,]<-mvrnorm(1,mu=c(mu1[j],mu2[j],mu3[j]),Sigma=sigma)

}

Y1<-data[,1]

Y2<-data[,2]

X<-data[,3]

data<-data.frame(Y1,Y2,X,M1,M2)

data$Y1[data$M1=="1"]<-NA

data$Y2[data$M2=="1"]<-NA

attach(data)

#ARGUMENTS FOR MICE:

m=10

maxit=10

method=c("normSens","normSens","","","")

#NARFCS models are

# Y1~Y2+X+M2+M1

# Y2~Y1+X+M1+M2

predictorMatrix<- diag(0, ncol(data))

rownames(predictorMatrix) <- names(data)

colnames(predictorMatrix) <- names(data)

predictorMatrix["Y1",c("Y2","X","M2")] <- 1

predictorMatrix["Y2",c("Y1","X","M1")] <- 1

predictorSens<- diag(0, ncol(data))

rownames(predictorSens) <- names(data)

colnames(predictorSens) <- names(data)

seed=123456786

#####ALGORITHM 1:

MSPmodels<-list(Y1~M1,Y2~M2)

MSPfamily=c("gaussian","gaussian")

Substantive<-Y1~X

Subfamily=c("gaussian")

#We will fix ranges for both the two CSPs at c(-10,0) taking points 1 unit apart

l<-c(-10,-10,NA,NA,NA)

u<-c(0,0,NA,NA,NA)

step<-c(1,1,NA,NA,NA)

#Entries for X, M1 and M2 set to NA as they are complete

parms<-list(list(NA),list(NA),list(NA),list(NA),list(NA))

#RUN ALGORITHM:

ans<-calibrationr.fn(MSPmodels,MSPfamily,Substantive,Subfamily,Intercept=FALSE,l,u,step,parms)

#####ALGORITHM 2:

#Arguments the same as with algorithm 2, except substantive model arguments

elicited<-c(-10,-10)

tol<-c(0.6,0.6)

#RUN ALGORITHM:

ans<-calibrationAAO.fn(MSPmodels,MSPfamily,elicited,l,u,step,tol,parms)

#####ALGORITHM 3:

#Calibrate CSP for Y1 with CSP for Y2 at -10

MSPmodels<-Y1~M1

MSPfamily<-"gaussian" #QUOTATIONS VITAL

elicited<--10 #Elicited MSP is -10

l<--20

u<-0

step<-5

tol<-0.1

parms=list(list(NA),list(-10),list(""),list(""),list(""))

# CSP for Y1 is set to NA, CSP for Y2 to -10.

# X, M1 and M1 are complete, entry is therefore list("")

#RUN ALGORITHM:

ans<-calibrationOAAT.fn(MSPmodels,MSPfamily,elicited,l,u,step,tol,parms)

###############PERFORMING SENSITIVITY ANALYSIS For Algorithm 1##################

#We give a short example of doing the analysis from the results of algorithm 1

#Assume the range of interest is (-10,-5) for the Msp for Y1

#and(-8,-4) for the MSP of Y2.

#####Graphical Methods

#Plot the Msps against the analysis estimates

#INSTALL PACKAGE 'scatterplot3d'

library(scatterplot3d)

#3d plot

scatterplot3d(ans$results[,"MSP 1"],ans$results[,"MSP 2"],ans$results[,"est"],type="l",

angle=30)

#The flat plane clearly indicates the 'one at a time' assumption holds

#Graph individually and mark areas of interest for MSP of Y1

#Graph for MSP of Y1

plot(ans$results[,"MSP 1"],ans$results[,"est"],type="l")

abline(v=-10,col="red")

abline(v=-5,col="red")

#As effect of interest is Y1~X, Msp for Y2 is less important

#NOTE:

#To apply a tipping point analysis, plot against ans$results[,"Pr(>|t|)"]

#####By Tabulation

#Obtain the set of results such that the MSPs lie within their elicited

#ranges

ans$results[ans$results[,"MSP 1"]>=-10 &

ans$results[,"MSP 1"]<=-5 &

ans$results[,"MSP 2"]>=-8 &

ans$results[,"MSP 2"]<=-4 ,]

# It may be worth looking at values that lie slightly outside the elicited ranges as well
